# Editorial: Modelling of intravascular drug delivery using nanocarriers

**DOI:** 10.3389/fddev.2025.1681622

**Published:** 2025-10-06

**Authors:** Panagiotis Neofytou, Nicolae-Viorel Buchete

**Affiliations:** ^1^ Thermal Hydraulics and Multiphase Flow Laboratory, Institute of Nuclear & Radiology Sciences & Technology, Energy & Safety, National Centre for Scientific Research “Demokritos”, Agia Paraskevi, Greece; ^2^ School of Physics, University College Dublin, Dublin, Ireland; ^3^ Institute for Discovery, University College Dublin, Dublin, Ireland

**Keywords:** drug delivery nanocarriers, intravascular drug delivery, nanoparticle protein corona, fluid-nanocarrier interaction modelling, computational fluid-particle dynamics, nanomaterials for cancer treatment, molecular modelling of nanoparticle coronas, stealth effect of nanocarriers

Nanocarrier-based drug delivery systems have seen tremendous developments in recent years, enabled by rapid advances in both nano-scale technologies and *in silico* modelling tools. The articles published in this Research Topic capture a snapshot of this rapidly developing area, focusing on computational modelling methods that complement recent experimental approaches to studying nanocarrier intravascular drug delivery (NIVDD) systems, as it has long been recognized that there is a need for the development of more standardized frameworks for *in silico* preclinical trials. Five papers are included in this Research Topic, and it is our hope that they can address this need by bringing together a broad range of recent approaches and highlighting their promises and limitations.

The first paper by Buchete et al. is a comprehensive review of both main historical events resulting in the development of NIVDD systems and the broad range of multiscale physics-based approaches employed by different research groups. The first section introduces nanoparticle (NP)-based drug delivery approaches, highlighting systematically ideas related to aspects such as NP types, methods for NP loading with useful drugs and concepts related to the effective *in vivo* targeting and biophysical interactions (i.e., that ultimately modulate delivery) of drug-carrying NPs with their cellular targets. The second section illustrates the primarily molecular modelling-based approaches involved in microscale and mesoscale modelling, illustrating the corresponding molecular-level concepts and implications, from NP-functionalization and loading stages to form a NIVDD system to the NC’s molecular interactions in the blood, including the formation of a protein corona (i.e., protein-rich biomolecular structures surrounding NPs), to the delivery stage when the desired targets (e.g., extracytosolic cellular membrane receptor surfaces) are finally reached. Notably, multiscale modelling approaches of complex molecular processes, such as the complex NIVDD coverage by proteins while located in the blood, are also illustrated and discussed. The third and final section highlights the larger, macroscale aspects of functionalized NIVDD systems, covering physical properties such as the convection and diffusion of nanoparticles in biological media in general, and in blood vessels and capillaries in particular. It is illustrated how models based on computational fluid dynamics (CFD) are needed and used to describe larger-scale processes such as particle-wall interactions in capillaries, related to triggering receptor-ligand reactions at vascular areas of interest. Ultimately, the CFD-based approaches can be both improved by the microscale molecular-level models presented in the second section and can also provide a useful benchmark themselves (i.e., in a multiscale feedback loop) for achieving improved accuracy at different scales in a self-consistent manner.

Another paper by Li et al. (Southeast University, Nanjing, China) presents recent developments in cancer treatment regarding the use of nanomaterials for iron homeostasis. Cells in highly vascularized tumor tissue exhibit a large increase in iron uptake, required for their pronounced growth, migration and possible invasion stages related to cancer proliferation. This paper illustrates the author’s experience with a novel class of nanocarriers using iron chelating agents such as deferoxamine (DFO), deferasirox (DFX) and Dp44mT, which showed promise in being used in conjunction with different types of nanoparticles. This approach can advance the current NIVDD toolbox of systems available to target selectively and efficiently cancer cells and tumors and could impair their proliferation by modulating their access to iron.

A third paper by Fuoco (Department of Chemical Engineering, Polytechnique Montréal, Montréal, QC, Canada) presents another interesting high-level view of using NPs as nanocarriers from a more user-facing perspective, providing not only a pragmatic summary of the recent concepts and key points involved in practical development of NIVDD systems, including pointers to different types of NP materials and nanotechnologies, but also including pointers to public and private-access databases and information sources that could be consulted for identifying recent developments in this field. Notably, the review highlights emerging biomedical aspects that need to be considered for improving the clinical outcomes of NIVDD systems, such as their “stealth effect” (i.e., the biophysical and biomolecular properties that can allow them to avoid recognition by the immune system and trigger their premature clearing). Other related aspects are also discussed, such as cellular or even molecular “Trojan-horse” strategies that may be used to improve both delivery efficiency and targeting specificity.

Finally, the research article by Koutsi et al. (Department of Mechanical and Manufacturing Engineering, University of Cyprus, Nicosia, Cyprus) introduces a novel complex mathematical modelling approach to study and quantify the combined effects of both mechanotherapy and sonopermeation on tumor treatment. The accurate modelling of a cancer tumor’s microenvironment (TME) can play a key role in modulating both its development and its response to various treatments (e.g., exposing the tumor to IV-delivered nanocarriers). This paper lays the foundations of developing an automatic and systematic modeling framework, including explicitly three principal states of nanocarrier-based drug delivery (i.e., (i) encapsulation/functionalization of the chemotherapeutic agent, (ii) free diffusion of the agent in the tumor interstitial space, and (iii) internalization for the chemotherapeutic agent by the cells), to model the more complex processes involving the combined use of mechano-therapeutics and sonopermeation and make connection to *in vitro* and, ultimately, clinically-relevant experiments.

We are grateful to all the authors, editorial assistants and reviewers who made this Research Topic possible. We hope that bringing together this expertise will provide both easily and openly accessible references for researchers working on developing modern NIVDD systems, will provide guidelines for the terminology used and the concepts that need to be taken into account in NIVDD design, and will inspire new studies at multiple scales that can improve specificity and selectivity and, ultimately, their overall biomedical efficacy.

